# Nuclear localization and cytosolic overexpression of LASP-1 correlates with tumor size and nodal-positivity of human breast carcinoma

**DOI:** 10.1186/1471-2407-7-198

**Published:** 2007-10-23

**Authors:** Thomas GP Grunewald, Ulrike Kammerer, Michaela Kapp, Matthias Eck, Johannes Dietl, Elke Butt, Arnd Honig

**Affiliations:** 1 Institute of Clinical Biochemistry and Pathobiochemistry, University of Wuerzburg, Grombuehlstr. 12, D-97080 Wuerzburg, Germany; 2 Department of Obstetrics and Gynecology, University of Wuerzburg, Josef-Schneider-Str. 4, D-97080 Wuerzburg, Germany; 3 Institute of Pathology, University of Wuerzburg, Josef-Schneider-Str. 2, D-97080 Wuerzburg, Germany

## Abstract

**Background:**

LIM and SH3 protein 1 (LASP-1), initially identified from human breast cancer, is a specific focal adhesion protein involved in cell proliferation and migration, which was reported to be overexpressed in 8–12 % of human breast cancers and thought to be exclusively located in cytoplasm.

**Methods:**

In the present work we analyzed the cellular and histological expression pattern of LASP-1 and its involvement in biological behavior of human breast cancer through correlation with standard clinicopathological parameters and expression of c-erbB2 (HER-2/neu), estrogen- (ER) and progesterone-receptors (PR). For this purpose immunohistochemical staining intensity and percentage of stained cells were semi-quantitatively rated to define a LASP-1 immunoreactive score (LASP-1-IRS). LASP-1-IRS was determined in 83 cases of invasive ductal breast carcinomas, 25 ductal carcinomas in situ (DCIS) and 18 fibroadenomas. Cellular LASP-1 distribution and expression pattern was visualized by immunofluorescence and confocal microscopy and assessed through separate Western blots of nuclear and cytosol preparations of BT-20, MCF-7, MDA-MB231, and ZR-75/1 breast cancer cells.

**Results:**

Statistical analysis revealed that the resulting LASP-1-IRS was significantly higher in invasive carcinomas compared to fibroadenomas (p = 0.0176). Strong cytoplasmatic expression of LASP-1 was detected in 55.4 % of the invasive carcinomas, which correlated significantly with nuclear LASP-1-positivity (p = 0.0014), increased tumor size (p = 0.0159) and rate of nodal-positivity (p = 0.0066). However, levels of LASP-1 expression did not correlate with average age at time point of diagnosis, histological tumor grading, c-erbB2-, ER- or PR-expression.

Increased nuclear localization and cytosolic expression of LASP-1 was found in breast cancer with higher tumor stage as well as in rapidly proliferating epidermal basal cells. Confocal microscopy and separate Western blots of cytosolic and nuclear preparations confirmed nuclear localization of LASP-1.

**Conclusion:**

The current data provide evidence that LASP-1 is not exclusively a cytosolic protein, but is also detectable within the nucleus. Increased expression of LASP-1 in vivo is present in breast carcinomas with higher tumor stage and therefore may be related with worse prognosis concerning patients' overall survival.

## Background

Breast cancer is the most frequent malignancy among women and ranks first as cause of cancer deaths among women at ages between 20 to 59 years [1]. Despite the use of endocrine therapy, systemic chemotherapy and novel approaches such as treatment with trastuzumab (Herceptin^®^), outcome of metastatic breast cancer has not substantially improved. Metastatic disease remains generally incurable with a median survival time of only a few years [2,3]. Thus, new therapeutic modalities are required to improve the outcome. Genes that are overexpressed in metastatic cancer cells are promising targets for novel therapeutic agents.

The LIM and SH3 domain protein LASP-1 was initially identified from a cDNA library of breast cancer metastases. The gene was mapped to human chromosome 17q21 in a region that is altered in 20–30% of human breast cancers [4,5], suggesting that it could play a role in tumor development and metastases of breast cancer.

Human LASP-1 encodes a membrane-bound protein of 261 amino acids containing one N-terminal LIM domain, followed by two actin-binding sites and a C-terminal src homology SH3 domain. The actin-binding domains in the core of LASP-1 mediate an interaction between LASP-1 and actin at cell membrane extensions, but not along actin stress fibers [6-10].

Recent data showed an additional interaction of LASP-1 via its nebulin like actin-binding repeats with kelch related protein 1 (Krp1), a focal adhesion protein involved in cell migration. The exact cellular function of LASP-1 is not known yet, but the protein has previously been reported to localize within multiple sites of dynamic actin assembly such as focal contacts, focal adhesions, lamellipodia, membrane ruffles and pseudopodia [4,7,11-13].

The C-terminal SH3 domain of LASP-1 is involved in protein-protein interactions through binding to proline-rich sequences, specifically with zyxin, palladin, lipoma preferred partner (LPP) and vasodilator stimulated phosphoprotein (VASP) [9,14,15]. Mutation analysis of LASP-1 led to the conclusion that its SH3 domain is necessary for pseudopodial extension and invasion [16].

Although no binding partner for the LIM domain of LASP-1 has been identified so far, previous data have shown that the zinc-finger module in the LIM domain of LASP-1 is an morphologically and perhaps functionally independent folding-unit of this protein harboring the possibility of direct binding to DNA [17].

Moreover, LASP-1 is substrate of Abelson tyrosine kinase. Abelson tyrosine kinase is strongly involved in carcinogenesis of hematopoetic tumors, such as B-cell lymphomas [18]. Phosphorylation of LASP-1 at tyrosine 171 is associated with loss of LASP-1 from focal adhesion points and the initiation of cell death, but without changes in dynamics of migratory processes [13]. In addition, phosphorylation of LASP-1 at serine 146 by cAMP- and cGMP-dependent protein kinases resulted in a translocation of the protein from membrane to cytosol and was followed by reduced cell migration [8]. All these protein-protein interactions mediated by the LIM and SH3 domains can be regarded as scaffolds for the formation of higher order complexes and suggest that LASP-1 could be part of important signaling pathways and a structural protein as well.

LASP-1 expression has been reported to be increased in metastatic breast cancer, suggesting that overexpression of LASP-1 may be involved in the migratory process of these cells [4]. Interestingly, knock-down of LASP-1 by RNA-interference in metastatic breast cancer cell lines BT-20 and MCF-7, as well as in the ovarian cancer cell line SKOV-3 resulted in a strong inhibition of proliferation, migration and in cell cycle arrest in G2-phase without induction of apoptosis or necrosis. Furthermore, LASP-1 silencing was accompanied by a reduced binding of the LASP-1 binding partner zyxin to focal contacts.

Reversely, artificial overexpression of LASP-1 in non-neoplastic PTK2 (Potorous tridactylis kidney) cells hardly expressing endogenous LASP-1, resulted in a acceleration of migration [19,20].

In this study we demonstrate that LASP-1 is not only a cytosolic, but also a nuclear located protein, which is highly overexpressed in breast cancer tissue compared to benign fibroadenomas. Furthermore, we provide evidence that its cytosolic overexpression and nuclear localization correlates significantly with tumor size and nodal-positivity of human breast carcinomas.

## Methods

### Tissue samples

The studies were performed with approval of the Ethics Committee of the University of Wuerzburg. Paraffin embedded tissue samples of 126 archival cases with confirmed histological diagnosis were obtained from the department of Pathology of the University of Wuerzburg.

We analyzed 25 cases of ductal carcinoma in situ without any invasive component (DCIS), 83 invasive ductal breast carcinomas and 18 fibroadenomas as well as three samples of normal breast tissue from reduction mammoplasty.

The patients with invasive breast carcinomas were aged from 32 to 96 (mean 58.6 ± 13.52) years. In this study all carcinomas, which were mainly collected from patients undergoing wide excisions, have been classified according to criteria of the WHO and recorded as invasive ductal carcinomas by a pathologist. Grading of malignancy of ductal carcinomas was evaluated according to the Scarff, Bloom and Richardson criteria with guidelines as suggested by Nottingham City Hospital Pathologists [21]. Tumor staging was performed according to parameters of the TNM system [22].

### Immunohistochemistry

For immunohistochemical staining procedures tissue sections were cut from regular paraffin embedded tissue at 2–3 μm. Sections were placed onto APES (3-amino-propyltriethoxy-silane; Roth, Karlsruhe, Germany) coated slides, dewaxed in xylene, rehydrated in graded ethanol and in TRIS-buffered saline (TBS; 25 mM TRIS/HCl, pH 7.4, 137 mM NaCl, 2.7 mM KCl). For antigen retrieval, sections were subjected to heat pretreatment by boiling it in 0.01 M of sodium citrate buffer (pH 6.0) for 10 min in a microwave oven (600Watt/sec.). Endogenous peroxidase was blocked by incubation in 0.1% hydrogen peroxide in PBS for 5 min. Slides were then incubated with the polyclonal anti-LASP-1 antibody [8] diluted 1:1000 in "antibody diluent" (DAKO, Hamburg, Germany) followed by EnVision/rabbit detection system (DAKO, Hamburg, Germany). 3,3'-Diaminobenzidine (DAB; DAKO, Hamburg, Germany) was used as chromogen and sections were counterstained in hematoxylin (Mayers, Sigma, Deisenhofen, Germany), dehydrated through graded ethanol and embedded in Entelan (Merck, Darmstadt, Germany).

### Evaluation of immunohistochemical LASP-1 staining and LASP-1-IRS

To assess the role of LASP-1 in human breast cancer, we examined its expression in 83 breast carcinoma samples from patients selected randomly from January 2000 to December 2006 with or without invasive components.

Semi-quantitative evaluation of LASP-1 immunostaining was carried out by three independent observers (TG, UK and EB) through defining of the percentage of positive cells and the staining intensity as described below. In most of all cases (> 90%) the independently determined LASP-1-IRS was consistent within all observers. In the rare event of divergent evaluation, a consensus was found. For positive controls we used breast cancer sections previously described as highly LASP-1-positive [19]. In negative controls with omitted primary antibody or with pre-immune serum no staining was observed.

Scoring of cytosolic LASP-1 expression was carried out in analogy to scoring of hormone receptor Immune Reactive Score (IRS) ranging from 0–12 according to Remmele et al. [23], which is used routinely in surgical pathology for the quantification of hormone receptor expression in mammary carcinoma.

**Figure 1:**
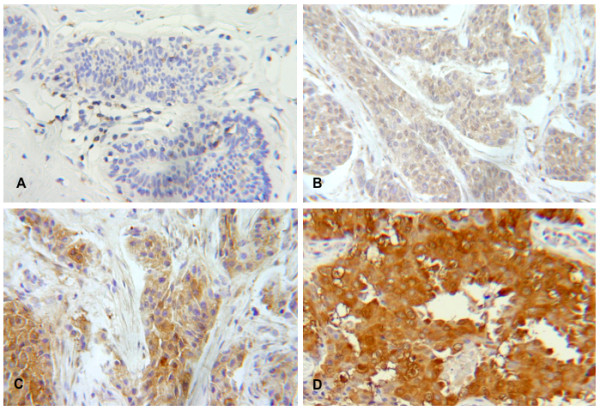
**Heterogeneous LASP-1 expression in human invasive breast cancer**. Immunohistochemical staining of different LASP-1 expression intensity levels in human invasive breast cancer samples (DAB, brown; magnification 100×). (A+B) low LASP-1-IRS (< 5). (C+D) medium to high LASP-1-IRS (> 5).

The percentage of LASP-1-postitive stained cells was scored in five grades (grade 0 = 0–19%, grade 1 = 20–39%, grade 2 = 40–59%, grade 1 = 60–79% and grade 4 = 80–100% LASP-1 expressing tumor cells). The fraction of LASP-1-positive stained cells was scored after having examined 10 high-power fields (40×) of one section for each sample. In addition, the intensity of LASP-1 expression by the tumor cells was determined (grade 0 = none, grade 1 = low, grade 2 = moderate, grade 3 = strong). The multiplication of these two grading scores calculates the immunoreactive score for LASP-1 expression (LASP-1-IRS) in stained tissue (% LASP-1-positive tumor cells × staining intensity = LASP-1-IRS). Examples for the very heterogeneous LASP-1 expression in invasive breast cancer are given in Figure 1.

For better statistical discrimination samples scored with cytosolic LASP-1-IRS < 5 were classified as LASP-1-negative, those with LASP-1-IRS > 5 or higher as LASP-1-positive.

Nuclear LASP-1-positivity was scored by determining percentage of positive nuclei regardless of cytosolic LASP-1 expression and staining intensity. In analogy to the scoring of the proliferation marker Ki67 samples were considered as nuclear-positive (NUC+) if 10% or more cells showed nuclear staining for LASP-1 [24].

The immunomarkers c-erbB2 (HER-2/neu), estrogen receptor (ER) and progesterone receptor (PR) assessed in this study had been previously detected by standard immunohistochemistry and were drawn from the archival database of the Department of Pathology, Wuerzburg.

### Statistical analysis

Graph Pad Prism software test statistics was used to assess LASP-1 expression and the categorical parameters of interest. Furthermore, multivariate non-parametric analysis was performed using Fisher's exact (F) and Mann-Whitney (M) test. In the statistical analysis invasive ductal carcinomas were sorted in groups depending on nodal-positive or nodal-negative status and small (T1 = ∅ max. 2 cm) or larger tumor size (> T1). This dichotomous graduation was made on the basis of a recent meta-analysis stating that the most beneficial prognostic criteria are nodal-negativity and a small tumor size at time point of diagnosis [25].

Further stratification of our tumor samples according to extent of nodal positivity and advanced tumor size was abandoned, since there are no additional major therapeutic implications [26].

### Cell culture conditions

Cell lines (MCF-7, BT-20, MDA-MB231 and ZR-75/1) were obtained from Cell Line Services (Heidelberg, Germany) and grown at 1 × 10^5 ^cells/ml in a plastic cell culture flask in a humidified incubator at 37°C under 5% CO_2 _atmosphere in HBCA-medium [27] containing 10% heat-inactivated fetal bovine serum (PAA, Linz, Austria) and 1% streptomycin/ampicillin (Invitrogen, Karlsruhe, Germany). Cells were cultured until homogeneous morphology of cells was reached (passage 3–4) since LASP-1 belongs to a group of several differential expressed proteins that are up-regulated after later passages [28].

### Immunofluorescence and confocal imaging

For confocal microscopy, cells were grown until homogenous morphology at a maximum of 70% confluence on glass chamber slides, fixed in 4% (w/v) paraformaldehyde in PBS, permeabilized with 0.1% (w/v) Triton X-100 in PBS, and then stained with affinity-purified LASP-1 antibody (1:2000) followed by secondary Cy3-labeled anti-rabbit antibody (1:500) (Dianova, Hamburg, Germany).

Fluorescence and transmission-DIC images were recorded on a modified confocal microscope (Leica SP5, Mannheim, Germany) with a 100× NA 1.4 objective (Leica, Wetzlar, Germany). Fluorescence was detected with SP5 spectral emission setting at 570–650 nm for the Cy3 and with the DIC-transmission channel. The images were recorded with 512 × 512 pixels with lateral resolution between 90 and 200 nm and a recording rate of 400 lines per second. Each image was also line averaged 4 times and the entire frame was averaged twice for optimal signal to noise ratio. The images were converted to .tiff format and analyzed with Photoshop™ software.

### Preparation of nuclear and cytosolic cell fractions

Human breast cancer cell lines were harvested at 80% confluence through trypsination. Isolation of nuclei and cytosol was carried out using NE-PER Nuclear and Cytoplasmic Extraction Reagents (Pierce, Bonn, Germany) following the manufacturers instructions. Probes were solved in Laemmli sample buffer at a final concentration of 1 × 10^6 ^/ml and stored at -20°C before Western Blot electrophoresis.

### Western blot analysis

For Western blotting cells were lysed in Laemmli sample buffer. Equal amounts of protein, according to cell count, were resolved by 12% SDS-PAGE. After blotting on nitrocellulose membrane and blocking with 3% nonfat dry milk in 10 mM Tris, pH 7.5, 100 mM NaCl, 0.1% (w/v) Tween 20, the membrane was first incubated with the antibody raised against LASP-1 (1:10000) [8] followed by incubation with horseradish peroxidase-coupled goat anti-rabbit IgG (Biorad, Munich, Germany), diluted 1:5000, and visualization was done using ECL (Amersham Biosciences, Freiburg, Germany). Protein bands were visualized by autoradiography. Quantification of autoradiography signals was carried out by densitometry using the ImageJ software (NIH, Bethesda, USA).

GAPDH was used as a specific cytosolic marker to exclude cytoplasmatic contamination of the nuclei preparation and was visualized by incubating NC membrane with polyclonal anti-GAPDH primary antibody (1:1000; Santa Cruz, Santa Cruz, USA). Anti Lamin A+C antibody (1: 50; Abcam, Cambridge, UK) served as a specific nuclear marker to exclude nuclear contamination in cytoplasmatic cell samples [29-31]. At least three independent experiments have been carried out and representative results are shown.

## Results

### LASP-1 is overexpressed in invasive breast cancer tissue

**Figure 2:**
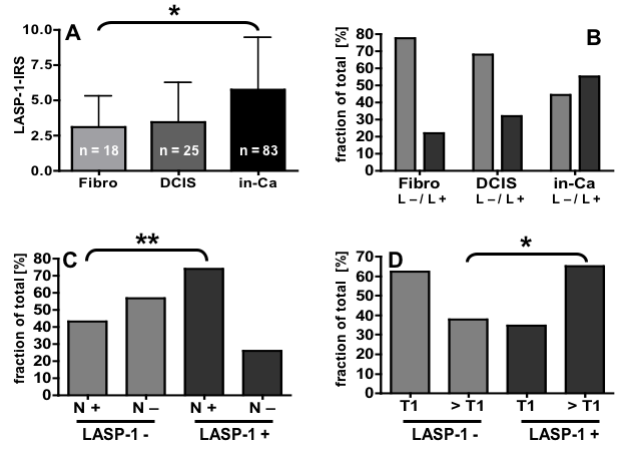
**Graphical illustration of statistical LASP-1 distribution.** (A) LASP-1-IRS (immunoreactive score) is significantly higher in invasive breast carcinomas (in-Ca) compared to fibroadenomas (Fibro). Error bars represent standard deviation. (B) Distribution of LASP-1-positivity (L+) and LASP-1-negativity (L-) in fibroadenomas, DCIS and invasive breast cancer. (C+D) Positive nodal status (N+) and tumor size (>T1) correlate significantly with LASP-1-positivity. Significant statistical differences are labeled with one star (p < 0.05) or two stars (p < 0.01).

LASP-1 expression was detected in cytoplasm of tumor cells, leukocytes, myoepithelial cells and vascular smooth muscle cells, but not in stromal cells. Immunohistochemistry clearly allowed to localize LASP-1 expression in carcinoma cells of 76 malignant breast carcinomas (91.56 %), whereas in seven patients LASP-1 could not be detected in invasive neoplastic cells. Medium to high LASP-1-IRS (>5) was observed in 46 cases (55.4%), which were considered to be LASP-1-positive, while 37 probes (44.6%) showed a low LASP-1-IRS (<5) and were considered to be LASP-1-negative (Table 1 and Figure 2B). In contrast, LASP-1 could not be detected in benign epithelial cells of reduction mammoplasty.

**Table 1:** Statistical analysis of LASP-1 expression in 83 breast cancer samples (in-CA), 25 ductal carcinomas in situ (DCIS) and 18 fibroadenomas (FIBRO). IRS: immune reactive score; n.s.: not significant

	**LASP-1 +**		**LASP-1 -**		**IRS mean**	**IRS STDV**	**IRS Median**	**p-values **(Fisher's exact)		
							
	**n**	**%**	**n**	**%**						
FIBRO	4	22.2	14	77.8	3.11	2.22	3	0.7315 n.s.		0.0176*
DCIS	8	32	17	68	3.48	2.80	3	0.7315 n.s.	0.066 n.s.	0.0176*
in-CA	46	55.4	37	44.6	5.75	3.73	8		0.066 n.s.	0.0176*

**Figure 3:**
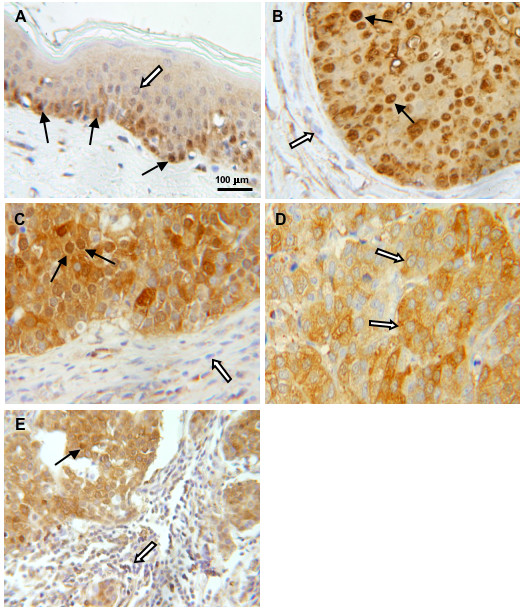
**Histological expression pattern of LASP-1.** Immunohistochemical staining of LASP-1 (DAB, brown, magnification 100×) in different cell types. White arrows indicate LASP-1-negative nuclei, black arrows LASP-1-positive nuclei. (A) LASP-1 is highly overexpressed in physiologically proliferating epidermal basal cells, compared to superficial epidermal strata or dermal connective tissue cells. (B+C) LASP-1 is localized abundantly in nuclei of invasive breast cancer cells compared to LASP-1-negative nuclei of stromal cells. (D) Invasive breast cancer cells with LASP-1-negative nuclei but perinuclear and cytosolic LASP-1 overexpression. (E) Nuclear and cytosolic LASP-1-positive breast cancer cells in direct neighborhood to infiltrating LASP-1-negative lymphocytes.

In analogy to previous findings in myoepithelial and vascular smooth muscle cells of human breast and ovarian tissue [19,20], LASP-1 overexpression could be observed in highly proliferating epidermal basal cells (Figure 3A), while cells of non-proliferating superficial layers or dermal connective tissue cells like fibroblasts showed only weak LASP-1 expression. Interestingly, strong nuclear LASP-1-positivity could be observed in about 29% of all breast carcinomas as well as in nuclei of epidermal basal cells (Figures 3A, 3B and 3C), whereas all other breast carcinoma nuclei were negative for LASP-1 and the cells only displayed perinuclear and cytosolic LASP-1 enrichment (Figure 3D).

In 89% of all tumor-samples, which were scored to be LASP-1-negative, LASP-1 was not detectable within the nucleus, while 43.5% of all LASP-1-positive specimens showed clear nuclear LASP-1 staining. Thus nuclear staining is correlated with cytosolic LASP-1 expression and significantly higher in LASP-1-positive cells compared to LASP-1-negative samples (p = 0.0014; Table 2).

**Table 2:** Statistical analysis of LASP-1 distribution and expression in correlation to clinicopathological and biological parameters. LASP-1 (L) protein expression was analyzed in 83 breast cancer samples. Associations with clinicopathological and biological parameters were analyzed using Mann-Whitney-test (M) and Fisher's exact test (F); n.s.: not significant.

		**Total **(n = 83)		**L + **(n = 46)		**L **- (n = 37)		**p-value **(Test)
			
		**n**	**%**	**n**	**%**	**n**	**%**	
Nodal status	N+	50	60.2	34	73.9	16	43.2	0.0066 (F)
	N-	33	39.9	12	26.1	21	56.8	**
Tumor size	T1	39	47	16	34.8	23	62.2	0.0159 (F)
	>T1	44	53	30	65.2	14	37.8	*
Grading	G1	3	3.6	0	0	3	8.1	0.9593 (M)
	G2	39	47	24	52.2	15	40.5	n.s.
	G3	41	49.4	22	47.8	19	51.4	
c-erB-2	Her+	18	21.7	12	26.1	6	16.2	0.3005 (F)
	Her-	65	78.3	34	73.9	31	83.8	n.s.
Estrogen receptor	ER+	60	72.3	33	71.7	27	73	1.0 (F)
	ER-	23	27.7	13	28.3	10	27	n.s.
Progesterone receptor	PR+	51	61.5	26	56.5	25	67.6	0.3673 (F)
	PR-	32	38.5	20	43.5	12	32.4	n.s.
Nuclear positivity	NUC+	24	29	20	43.5	4	11	0.0014 (F)
	NUC-	59	71	26	56.5	33	89	**

However, two samples (2.4%) showed a very high nuclear LASP-1-positivity with concurrent low cytosolic staining (Figure 3B).

This nuclear staining is unlikely to be unspecific, because nuclei of other benign stromal cells like fibrocytes are LASP-1-negative, even when located right next to cancer cells (Figures 3B and 3C).

This observation is even more obvious in Figure 3E, showing strong LASP-1-positive nuclei and cytosol of human breast cancer cells in comparison to LASP-1-negative nuclei of neighboring infiltrating lymphocytes.

### LASP-1 expression is significantly higher in invasive breast cancer compared to fibroadenomas and is correlated with TNM-staging

Comparison of average LASP-1 expression of fibroadenomas, DCIS and invasive breast cancer demonstrated that invasive cancer cells display significantly higher LASP-1 expression than fibroadenomas (p = 0.0176), as seen in Table 1 and Figure 2A. In contrast, staining intensity of LASP-1 in DCIS was not significantly higher than in fibroadenomas, but also not significantly lower than in invasive breast cancer (Table 1 and Figures 2A and 2B).

To evaluate the clinical relevance of the heterogeneous LASP-1 expression, LASP-1-IRS was compared to clinicopathological and biological parameters. Positive correlations were found between LASP-1-IRS and TNM-staging regarding tumor size T (p = 0.0159) and nodal-positivity (p = 0.0066; Table 2) (Figures 2C and 2D). No correlation was found with age at time of surgery (Table 3), grading, ER- and PR-positivity and HER-2/neu-expression (Table 2).

**Table 3:** Statistical analysis of LASP-1 (L) expression in relation to age. FIBRO: fibroadenome (n = 18); DCIS: ductal carcinomas in situ (n = 25); in-CA: invasive breast cancer (n = 83).

	**FIBRO**		**DCIS**		**in-CA**	
	
	**L +**	**L -**	**L +**	**L -**	**L +**	**L -**
Age (yrs) mean	40.8	40	54.1	54.5	61.1	55.1
STDEV	8.9	16.1	11.6	13.7	13.7	11.9

To evaluate the possible relevance of LASP-1 as a prognostic marker for lymph node metastasis in human breast cancer disease, a contingency test was performed and prognostic indices were calculated. Sensitivity of LASP-1-IRS-scoring to predict node-positivity is 85% with a specificity of 36.4%, (positive and negative predictive value 73.9 vs. 51.3%, respectively).

### LASP-1 is detectable within the nucleus by confocal microscopy

**Figure 4:**
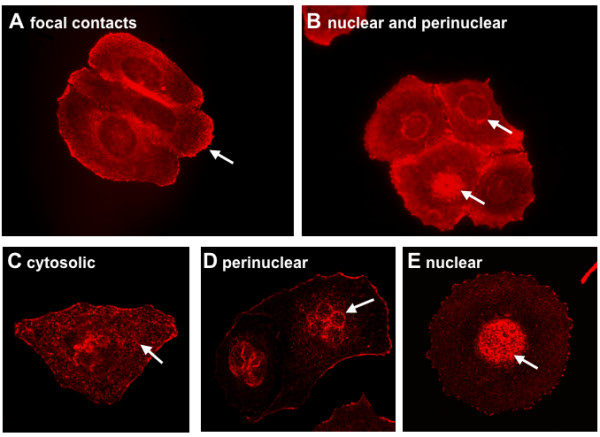
**Cellular LASP-1 expression pattern visualized by confocal microscopy**. Non-confocal (A+B) and confocal (C-E) microscopy of LASP-1 immunostaining in BT-20 breast cancer cells revealed that LASP-1 (red) is mainly detectable in focal contacts (A) and in the cytosol (C). In addition, more than 30% of the cells show a nuclear staining (B+E), and in some cells a perinuclear localization of LASP-1 is visible (B+D).

To further assess the cellular expression pattern of LASP-1, we performed confocal and non-confocal microscopy of immunofluorescence labeled LASP-1 in the breast cancer cell line BT-20. The immunofluorescence images showed a variable cellular expression pattern of the protein. In addition to the reported localization of LASP-1 (white arrows) to focal contacts and tips of lamellipodia (Figure 4A) [4,7,11-13], LASP-1 was detected in the cytosol (Figure 4C), perinuclear (Figures 4B and 4D) and nuclear (Figures 4B and 4E). This is in accordance to the LASP-1 localization observed in the breast tissue samples (Figure 3). A similar immunohistochemical staining pattern was detected in MCF-7 breast cancer cells and SKOV-3 ovarian cancer cells (data not shown).

### LASP-1 is detectable in nuclear fractions of various breast cancer cell lines by Western blotting

**Figure 5:**
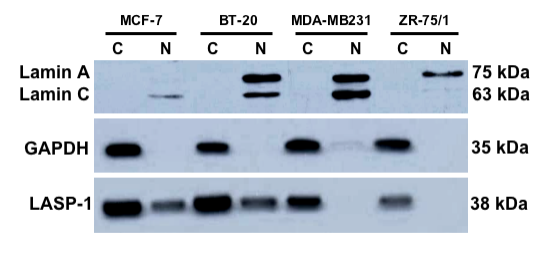
**Western Blot of nuclear and cytosolic fractions of the cancer cell lines MCF-7, BT-20, MDA-MB231 and ZR-75/1**. LASP-1 is detectable in nuclear (N) as well as in cytoplasmatic (C) cell fractions of breast cell lines MCF-7 and BT-20, but not in the nucleus of MDA-MB231 and ZR-75/1 cells. GAPDH was used as a cytoplasmatic marker, Lamin A + C as nuclear markers to exclude contamination during cell fraction isolation.

To verify the nuclear localization of LASP-1, MCF-7, BT-20, MDA-MB231 and ZR-75/1 breast cancer cells were separated in cytosolic and nuclear fractions and assessed by Western blot. Equal amounts of protein, according to cell count, were resolved by 12% SDS-PAGE and blotted on nitrocellulose membrane. As seen in Figure 5, LASP-1 is clearly detectable in the nuclei of human breast cancer cell lines MCF-7 and BT-20, while nuclei of MDA-MB231 and ZR-75/1 cells were found to be negative for LASP-1 in the nuclear fraction. GAPDH was used as a specific cytosolic marker to exclude cytoplasmatic contamination of nuclei samples during preparation. Reversely Lamin A+C served as a specific nuclear marker to exclude nuclear contamination in cytoplasmatic cell fractions (Figure 5).

## Discussion

In the present work we investigated for the first time the expression of LASP-1 in a series of 83 invasive breast carcinomas at protein level and compared the data to clinically established breast cancer parameters. We found that the degree of immunohistochemical staining correlated significantly with nodal metastasis and tumor size but seems to be independent of other parameters such as age, grading and estrogen or progesterone receptor status.

In contrast to earlier publications, demonstrating the co-amplification of the LASP-1 gene together with HER-2/neu (c-erbB2) [4,32], our statistical analysis revealed no relation between LASP-1 protein level and HER-2/neu protein expression.

A previous study also showed that LASP-1 mRNA is overexpressed in only 8–12% of all human breast cancers [5]. However, our immunohistochemical analysis provide evidence that the LIM and SH3 domain protein is highly expressed (LASP-1-positive) in 55.4% of all tested breast cancer samples. This discrepancy could be due to the fact that Tomasetto et al. [4] used total surgical specimens for their mRNA isolation, containing undefined amounts of LASP-1 free benign tissue, while our data focused on malign cells only.

Consistent with the high expression of LASP-1 in breast tumors recent data demonstrated the functional significance of LASP-1 for cancer metastasis. Silencing of LASP-1 by RNAi in highly LASP-1 expressing human breast and ovarian cancer cells led to reduced cell proliferation, migration and to cell cycle arrest in G2-phase [19,20].

These experiments are supported by our present study proving the significantly higher expression of LASP-1 in invasive breast carcinomas compared to benign fibroadenomas. The rate of strongly LASP-1 expressing samples of invasive ductal carcinomas amounted 55.4% (LASP-1-IRS > 5). Moreover, statistical analysis provided evidence for a positive correlation of cytosolic as well as nuclear LASP-1-positivity with tumor size and nodal-positivity indicating an important role of LASP-1 in proliferation and migration.

These data resemble those of another focal adhesion protein, ENAH. ENAH is a member of the ENAH/VASP protein family, which regulates cell migration and actin-cytoskeleton organization at focal contacts. Like LASP-1, ENAH is not detectable in benign breast epithelium, but is weakly expressed in low-risk benign diseases like fibroadenomas and strongly expressed in invasive breast cancers. Similar to LASP-1, there is a significant correlation of ENAH-expression and tumor size (p < 0.05) [33]. In contrast to LASP-1, siRNA induced ENAH-knock-down does not affect cell proliferation while LASP-1 silencing resulted in strong inhibition of cell growth and migration [19]. Thus, it is likely that among several focal adhesion proteins which are overexpressed in breast cancer LASP-1 has more regulative function than others.

In a recent investigation, a cDNA microarray was used to establish a prognostic index for nodal-positive breast cancer [34]. Similar to our study, all 20 patients were LASP-1-positive, albeit LASP-1 was found to be one out of five genes being under-expressed in patients that died within 5 years after surgery. This is in part differing from our results demonstrating a correlation between high LASP-1 protein levels and nodal-positivity. However, in many cases there are significant discrepancies between the measured mRNA levels and protein data indicating post-transcriptional mechanism of regulation and stabilization [35].

In the present work we provide evidence for LASP-1 being not only a cytosolic, but also a nuclear protein. By our immunohistochemical stainings LASP-1 was detectable in nuclei of 29% of all investigated breast carcinomas independent of its actual cytosolic expression. Images taken with confocal microscopy confirmed nuclear localization of LASP-1 within the nucleus in BT-20 and MCF-7 breast cancer cells. Although these monoclonal cell lines are genetically identical, immunostaining demonstrated a variable cytosolic and nuclear LASP-1 localization, possibly dependent on cell cycle. Western blot analysis verified nuclear LASP-1 localization in MCF-7 and BT-20 cells while breast cancer cell lines MDA-MB231 and ZR-75/1 only displayed a cytosolic but no nuclear LASP-1 localization. Cell line ZR-75/1 is known to be highly estrogen and progesterone dependent [36] and was found to have the highest c-erbB2-expression among eight characterized breast- and four ovarian-cancer cell lines [37], whereas MDA-MB231 cells are ER- and PR-receptor negative and express HER-2/neu only at very low levels [38]. However, in our study LASP-1-IRS as well as nuclear LASP-1-positivity did not correlate with ER-, PR- or HER-2/neu-expression.

Previous data have shown that the zinc-finger containing LIM domain of LASP-1 is a morphologically and perhaps functionally independent folding-unit offering the possibility of direct binding to DNA [17]. In general, LIM domains are specialized double zinc-finger motifs interacting with many different proteins in association with the cytoskeleton and even form homeodomains to become nuclear transcription factors [39,40]. No hetero- or homodimerization of LASP-1 has been reported yet [9]. However, LASP-1 binding partner zyxin is a LIM domain containing protein known to be a nuclear shuttle protein involved in cell migration and cell cycle control [41,42], which could act as a potential interaction partner of LASP-1 in cell core.

In our present study we could demonstrate that LASP-1 is not only highly expressed in fast proliferating malignant tumor cells, but also in proliferating regenerative epidermal basal cells, while slowly proliferating dermal fibroblasts are LASP-1-negative. This observation is consistent with previous findings showing that LASP-1 expression positively influences tumor cell proliferation [19,20]. However, preliminary results show no correlation between the well-known proliferation marker Ki67 and LASP-1 expression (data not shown). Nevertheless, previous publications have shown that Ki67 expression is often considered as false positive and is inferior in evaluating tumor proliferation activity compared to standardized mitotic index at optimal cut-off points. This implies that evaluation of patients' prognosis by Ki67 expression has to be appraised with caution [43].

Further statistical calculations with contingency tests demonstrated that according to our data, postoperative relevance of LASP-1 expression for prediction of nodal-positivity has a sensitivity of about 85%, suggesting that LASP-1 could be used as a predictive marker for lymph node metastasis together with other markers like the superior method of sentinel lymph node biopsy with an average sensitivity of about 95% [44]. Thus postoperative non-invasive LASP-1 scoring in primary tumor tissue could accomplish node-positivity prediction as an additional marker to invasive sentinel node biopsy, especially in cases of negative sentinel node biopsy or if patients reject invasive sentinel node biopsy.

## Conclusion

This study is the first description of LASP-1 as a nuclear protein, whose cytosolic expression and nuclear localization correlates in vivo with tumor size and nodal positivity of human invasive ductal carcinoma of the breast. In summary, our observations suggest an expanded role for LASP-1 in biological breast cancer behavior. Further prospective studies will be necessary to define the potential of LASP-1 as an independent marker for diagnosis of cancer as well as a marker for prognosis of this disease.

## Competing interests

The author(s) declare that they have no competing interests.

## Authors' contributions

TG, UK and AH drafted and wrote the manuscript, designed and coordinated the study, evaluated the immunohistochemical data and performed statistical analyses, EB participated in its design and carried out immunofluorescence and confocal microscopy, MK carried out the immunohistological stainings and Western blots, ME provided histopathological and immunohistological guidance, JD participated in the design of the study and provided patients' data. All authors read and approved the final manuscript.

## Pre-publication history

The pre-publication history for this paper can be accessed here:


